# Impact of Electrocautery on Fatigue Life of Spinal Fusion Constructs—An In Vitro Biomechanical Study

**DOI:** 10.3390/ma12152471

**Published:** 2019-08-03

**Authors:** Haidara Almansour, Robert Sonntag, Wojciech Pepke, Thomas Bruckner, Jan Philippe Kretzer, Michael Akbar

**Affiliations:** 1 Clinic for Orthopedics and Trauma Surgery, Heidelberg University Hospital, 69118 Heidelberg, Germany; 2 Laboratory of Biomechanics and Implant Research, Clinic for Orthopedics and Trauma Surgery, Heidelberg University Hospital, 69118 Heidelberg, Germany; 3 Institute of Medical Biometry and Informatics, University of Heidelberg, 69118 Heidelberg, Germany

**Keywords:** electrocautery, titanium alloy, cobalt-chrome alloy, fatigue behavior, biomechanical study

## Abstract

Instrumentation failure in the context of spine surgery is attributed to cyclic loading leading to formation of fatigue cracks, which later propagate and result in rod fracture. A biomechanical analysis of the potential impact of electrocautery on the fatigue life of spinal implants has not been previously performed. The aim of this study was to assess the fatigue life of titanium (Ti) and cobalt-chrome (CoCr) rod-screw constructs after being treated with electrocautery. Twelve spinal constructs with CoCr and Ti rods were examined. Specimens were divided into four groups by rod material (Ti and CoCr) and application of monopolar electrocautery on the rods’ surface (control-group and electrocautery-group). Electrocautery was applied on each rod at three locations, then constructs were cyclically tested. Outcome measures were load-to-failure, total number of cycles-to-failure, and location of rod failure. Ti-rods treated with electrocautery demonstrated a significantly decreased fatigue life compared to non-treated Ti-rods. Intergroup comparison of cycles-to-failure revealed a significant mean decrease of almost 9 × 10^5^ cycles (*p* = 0.03). No CoCr-rods failed in this experiment. Electrocautery application on the surface of Ti-rods significantly reduces their fatigue life. Surgeons should exercise caution when using electrocautery in the vicinity of Ti-rods to mitigate the risk of rod failure.

## 1. Introduction

Electrocautery and spinal implants are principal tools in the spine surgeon’s armamentarium. These tools are utilized in complex surgeries to treat a multitude of spinal conditions and to ameliorate patients’ disabilities [1,2].

Technological advancements in biomechanics and biomaterials have revolutionized the sphere of spinal instrumentation, enabling surgeons to widen the indications for corrective surgery, and broadened the array of surgical techniques. In this context, spinal fusion was first introduced by Hibbs and Peltier to stabilize a spine affected by tuberculosis [3,4]. Albee used the tibia as graft material for spine stabilization [5]. King [6] and Lange [7] attempted internal fixation [1,4]. Harrington [8] introduced an innovative rod distraction system to correct coronal deformity [4], and Cotrel and Dubousset metamorphosed the profession with their segmental instrumentation for simultaneous correction of coronal and sagittal planes, thereby facilitating a three-dimensional (3D) approach to treat a 3D spine deformity [9]. These surgeries demand sophisticated technical aptitude and hence require meticulous preoperative planning. Consequently, one of the major preoperative decisions is choosing the correct deformity-specific, pathology-appropriate, spinal fusion construct [1].

Spinal implants must fulfil many criteria to be considered safe and efficient. Qualities such as biocompatibility, osseointegration, high strength, low Young’s modulus, high corrosion, and wear resistance are pivotal to implants’ long-term performance. Because no current material can fulfil all the clinical and biomedical requirements, material scientists have endeavored to enhance these qualities utilizing many surface modification techniques [10]. These processes include mechanical treatment, sol-gel application, thermal spraying, chemical and electrochemical treatment, micro-arc oxidation, laser surface modification, friction stir processing, and ion application [11,12].

Heating techniques and plasmochemical techniques occupy an important role in the sphere of biomedical engineering and materials science. Heat treatment is utilized to increase the fatigue strength of an alloy and to orchestrate an optimal balance of the material’s ductility, machinability, and stability [10]. Plasma implementation is non-toxic because it is free of solvents [13]. Implementing techniques such as plasma assisted microwave chemical vapor deposition, plasma etching, plasma nitriding, and deposition of a-DLC (amorphous diamond-like carbon) layers inoculated with nitrogen and silicon have been shown to have a significant impact on the microstructure and surface characteristics of alloys [13].

Accurate and appropriate surface modification techniques diversify and optimize the clinical use of alloys in surgical fields. However, spine surgeries are overburdened with post-operative complications [2], including implant-related failure and rod fracture (RF) [1]. The overall complication rates of surgical corrections of spine-deformity are reported to be 30% within two-years after surgery [14,15,16,17,18]. An important source of complications remains the intrinsic limitation of the robustness of spinal implants [15,19,20,21,22,23,24]. In a prospective multicenter assessment of risk factors for early RF following adult spinal deformity (ASD) surgery with one-year follow-up, Smith et al. [16] identified that RF occurred in as many as 9% of ASD patients, and up to 22% in patients who underwent pedicle subtraction osteotomy (PSO), a powerful technique for the correction of sagittal spino-pelvic malalignment. In another retrospective study, Smith et al. [23] found that most RF occurred within one year of index surgery.

In the literature to date, several risk factors for RF have been analyzed. They could be patient-related—e.g., higher Body Mass Index (BMI), patient age, history of spine surgery, development of pseudarthrosis, and greater baseline sagittal spinopelvic malalignment (Sagittal Vertical Axis (SVA), Pelvic Tilt (PT), and Pelvic Incidence-Lumbar Lordosis (PI-LL) mismatch), and a greater need for sagittal correction—or implant-related (implant material, or in situ rod contouring in the context of PSO) [16,21,23,24]. Notably, the arthroplasty literature provided a clue to an underlying confounder camouflaged behind the subtleties of RF: the thermal damage of electrocautery to the microstructure of implants. Huber et al. [25] were the first to document a case of shaft breakage of a hip endoprosthesis secondary to contact with an electrocautery device. Subsequently, Konrads et al. [26] reported on four similar cases which occurred after revision surgery. These authors argued that the thermal microstructural disruption ensued from an intra-operative electric arc seen post-contact of the electrosurgical electrode with the implant; this could have eventually led to breakage of the implant at the contact point. In addition, Yuan et al. [27] examined 1859 explanted hip implants and concluded that “iatrogenic arc melting” due to electrocautery was responsible for the color changes and corrosion on the surface of the studied implants. Furthermore, via fatigue testing, metallurgic analysis, and electron microscopy, Sonntag et al. [28] delineated a microstructural change in the Ti6A14V base material with high-frequency electrosurgery on hip endoprosthesis, resulting in a decreased load-to-failure when the electrocautery tip was applied to high-load areas of the stem during revision surgery.

Clinically speaking, it is difficult to avoid contact between the electrocautery tip and the implant during spine surgery. Particularly in cases of revision surgery, these electrical arcs could be deemed common. 

To the best of our knowledge, this is the first biomechanical study that addresses this topic in spine surgery. In concordance with the American Standard for Testing and Materials, our objective was to assess the impact of electrocautery contact on the fatigue life of titanium (Ti) and cobalt-chrome (CoCr) spinal implants.

## 2. Materials and Methods

### 2.1. Spinal Fusion Constructs and Vertebrectomy Models

Twelve unused specimens of CoCr and Ti implants were included (Expedium Spine, DePuy, Raynham, MA, USA). All rods and pedicle screw constructs had the same diameter (5 mm) and were cut to a length of 100 mm. Monoaxial pedicle screws (Ti6-Al4-V, 5.5 mm × 7 mm × 30 mm) and locking screws (Ti6-Al4-V, 7 mm) were used to rigidly couple the rods on the vertebrectomy model according to manufacturer-specific instructions using original instruments. Models were composed of dual Polypoxymethylene (POM) with dimensions as described by the American Standard for Testing and Materials (ASTM) F1717 [29]. This standard provides a uniform approach to conduct fatigue testing on spinal implants. The two blocks comprising each vertebrectomy model simulate two adjacent vertebrae fixed by posterior-instrumentation. The standard distance between the two blocks was 56 mm.

Specimens were divided into four groups by rod material and application of monopolar-electrocautery device on the rods’ surface (Table 1). 

### 2.2. Electrocautery Contact

**Figure 1:**
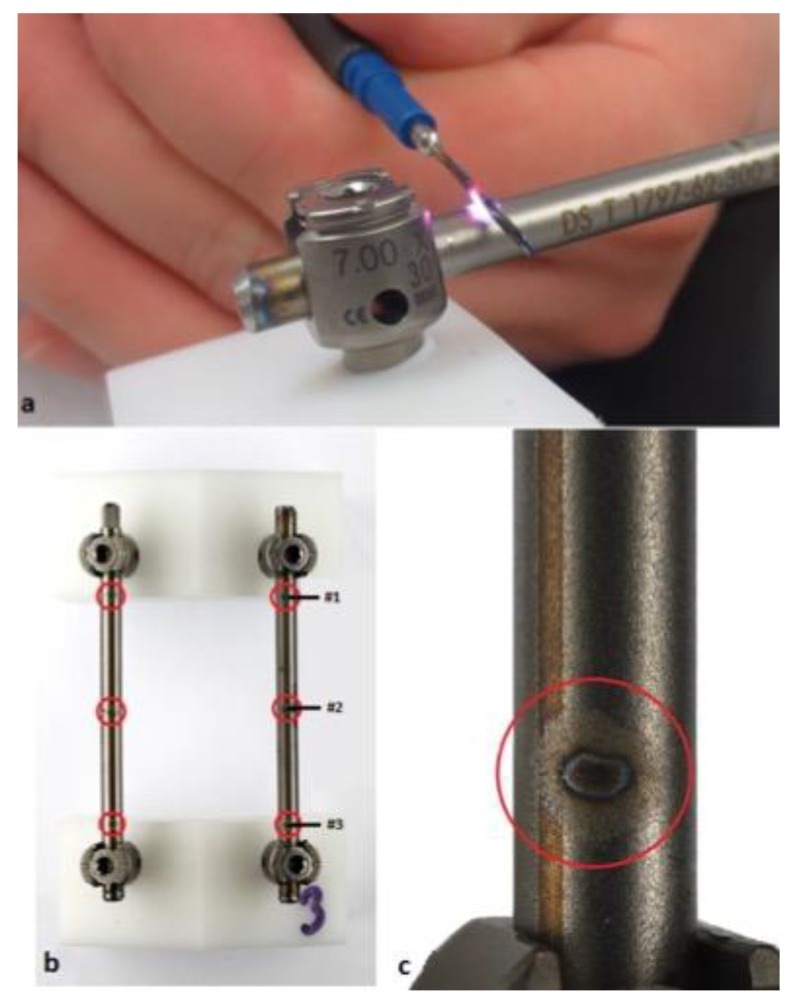
Electrocautery application. (**a**) Electric light arc, (**b**) sites, and (**c**) magnified view of the surface impact post electrocautery application. The first site was approximately 5 mm from the cephalic pedicle screw (#1). (#2 was at the center and #3 was analogous to #1 on the other side).

Electrocautery application (VIO-300, Erbe Elektromedizin, Tübingen, Germany) was performed by an experienced spine surgeon. Analogous settings used in the operation room of our institution were utilized (mode: swift-coag, 90 Watt, effect: 3, duration: ~4 s). High-frequency monopolar-electrocautery was applied on the surface of each rod at three defined positions (Figure 1). Under these conditions electric light arc was visible in all cases and discoloration of the rod surface was observed. 

### 2.3. Dynamic Mechanical Testing

**Figure 2:**
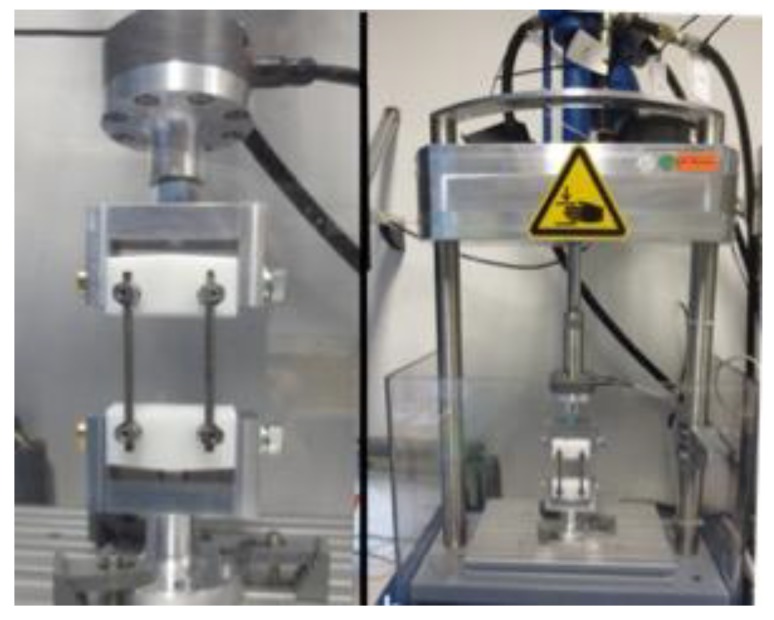
Frontal view of the fatigue testing setup.

Models were mounted in a servo-hydraulic uniaxial testing apparatus (HCE, Bosch-Rexroth, Lohr a. Main, Germany) under dry conditions such that the rods were aligned in the direction of vertical force application. The upper and lower vertebrae blocks were free to rotate in order to account for any bending of the rods during dynamic testing. A multistep fatigue test was performed at a frequency of 12 *Hz* (Figure 2). 

Tests started at an initial maximum sinusoidal load of 100 *N* (minimal load: 10 *N*). After one million cycles, the maximum load was increased by 50 *N*, and the load ratio *R* was kept constant at 0.1, until construct failure occurred. Based on the total number of steps and cycles completed, the estimated load-to-failure was calculated using this formula:

(1) $$F_{D}=F_{-1}+50N\cdot \frac{n}{1,000,000}$$

where $F_{-1}$ is the maximum force of the previous step before fracture, and n is the number of cycles in the step where the specimen failed.

### 2.4. Outcome Measures

Primary outcomes estimated load-to-failure, total number of cycles to failure, and location of rod failure.

### 2.5. Statistical Analysis

One-way analysis of variance (ANOVA) with post-hoc-test correction of least statistical difference was used to compare continuous values of the outcome measures. All data are presented as mean values ± standard deviation and 95% confidence interval. Statistical tests were computed using statistical package (SPSS-v.24, IBM, Armonk, NY, USA). The threshold of statistical significance was set at 0.05.

## 3. Results

Results of the biomechanical test are summarized in Table 2 and Table 3.

### 3.1. Titanium Control Group (Ti-CG) 

**Figure 3:**
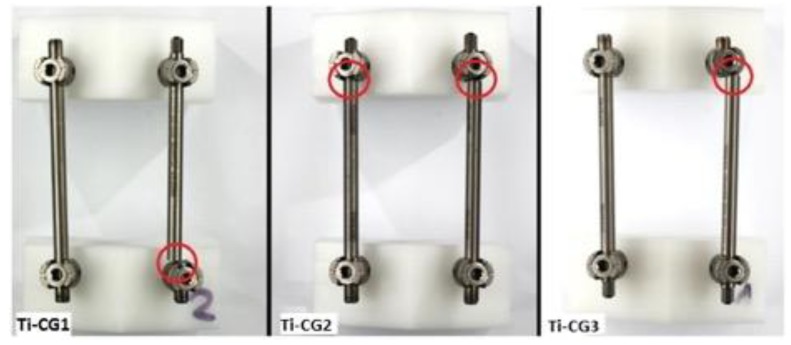
Sites of rod fracture of titanium control group (Ti-CG) post biomechanical testing and fatigue fractures of the rods at the rod-screw junction.

All specimens exhibited fatigue fracture of the rod at the rod-screw junction (Figure 3). 

### 3.2. Titanium Electrocautery Group (Ti-EG)

**Figure 4:**
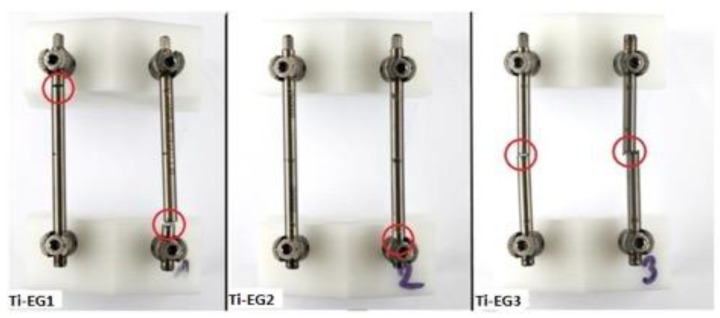
Sites of rod fracture of titanium electrocautery group (Ti-EG) post biomechanical testing.

In two specimens (Ti-EG1 and Ti-EG3), failure sites corresponded to the sites of electrocautery application (Figure 4). 

**Figure 5:**
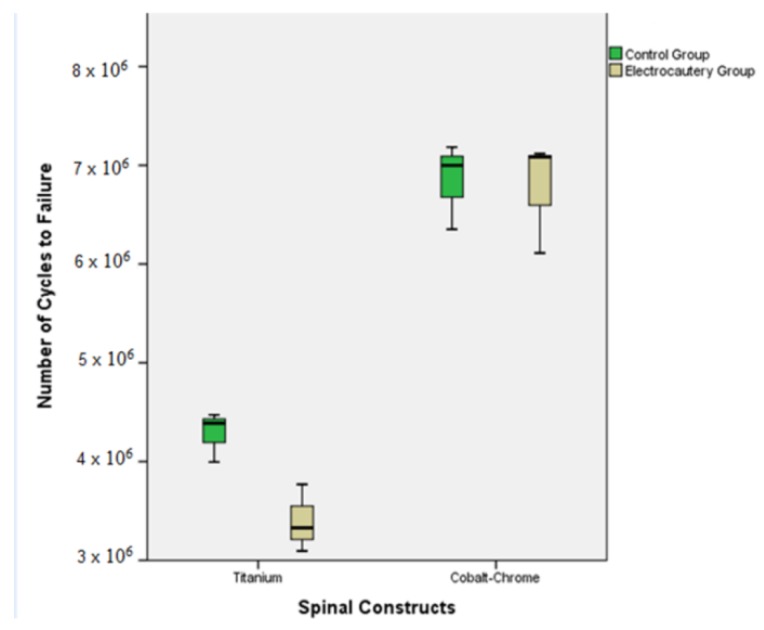
Boxplot representing the mean and 25% and 75% interquartile range of the total number of cycles to failure among the four tested groups.

Ti-EG revealed a significantly lower load-to-failure than Ti-CG (17% decrease, *p* = 0.023). Intergroup comparison of cycles to failure revealed a significant mean decrease of almost 9 × 10^5^ cycles (*p* = 0.03). (Figure 5). 

**Figure 6:**
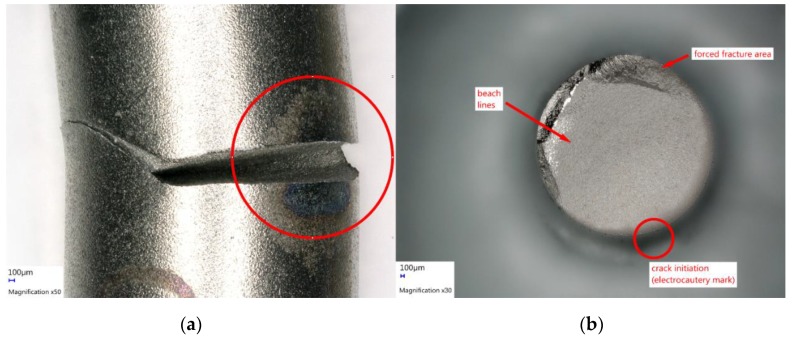
Microscopic analysis of fractured titanium rod after electrocautery (VHX-5000, Keyence, Osaka, Japan). (**a**) Post-fracture situation at the electrocautery mark. (**b**) Fracture surface with typical signs of fatigue fracture (beach lines and forced fracture area). The circle shows the location of crack initiation at the electrocautery mark.

Fatigue failure was confirmed by microscopic analysis of the fractured surface (Figure 6).

Notably, Ti-EG2 failed at the rod-screw junction similar to all of the Ti-CG rods.

### 3.3. CoCr Control Group (CoCr-CG) and Electrocautery Group (CoCr-EG)

In all tested constructs, the Ti pedicle screw failed. None of the CoCr-CG or CoCr-EG rods had fractured, exhibiting an increased load-to-failure in comparison to their Ti counterparts. 

**Figure 7:**
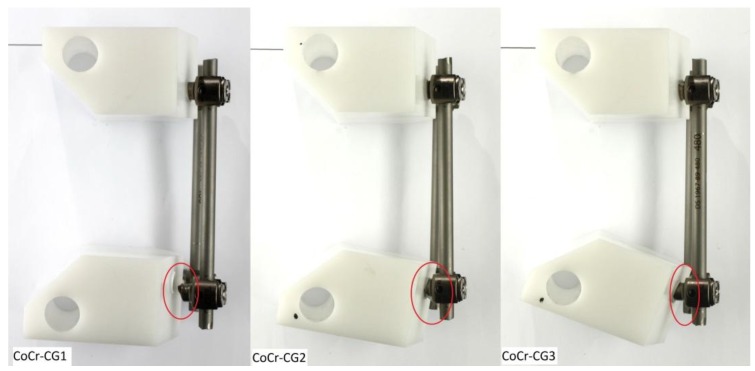
Sites of the titanium screw fracture of CoCr control group (CoCr-CG) post biomechanical testing.

**Figure 8:**
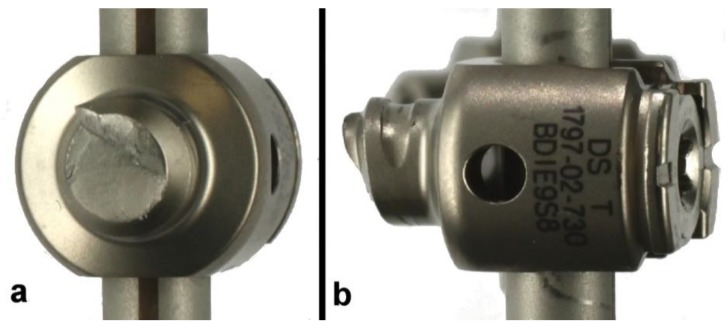
Magnified frontal (**a**) and lateral (**b**) views of the titanium screw fracture of one of CoCr electrocautery group constructs (CoCr-EG 3).

Intergroup differences were insignificant across all outcome measures. This implies that, in these tested constructs, the pedicle screw proved to be the weakest component under cyclic loading. In all specimens of these two groups, fatigue fracture transpired at the first thread of the titanium pedicle screw (Figure 7 and Figure 8).

## 4. Discussion

The human body is an intricate biomechanical composite that exerts cyclic loading on spinal instrumentation, which could lead to their failure [21,30]. The predominant use of electrocautery in operating-rooms mandates spine surgeons and implant engineers to contemplate the potential negative impact of electrocautery on the long-term integrity of spinal fusion constructs. Thus, addressing differences between the mechanical properties of these materials and studying the thermal effect of electrocautery are essential to predict the in vivo behavior of implants.

The micro-architecture of Ti and CoCr materials dictates their biomechanical performance and fatigue life [21,22,31]. Instrumentation failure is thought to be caused by recurring loading which generates oscillating stresses that may be well below the yield stress of the material. Unfortunately, this process is unnoticeable, either by the patient during daily activities or by the surgeon throughout follow-up visits, until final construct failure develops [21,32,33].

In vivo loading on spinal implants has been previously measured and reported to be 250 N in the prone position, 400 N whilst walking, and up to 700 N during exercise [34,35,36,37]. Notably, Ti-EG constructs failed at a maximum load of 250 N. This underlines the potential detrimental impact of electrocautery on the safety of Ti constructs, even within daily physiological loading. 

### 4.1. Thermal Damage and Notch Sensitivity: A Threat to Ti Biomechanical Integrity

Previous investigations have emphasized that the load-to-failure is contingent upon a material’s resistance to surface alterations. Fatigue fractures almost always nucleate at the exact location of notches or “discontinuity of geometrical structure” on the material’s surface [21,24,31,38,39]. This is particularly important when the site of the notch corresponds to areas where maximal loading is applied. Very small cracks relative to the dimensions of the micro-structure have been shown to cause failure faster than a long crack [40,41]. Therefore, the radius of the notch is, as a geometrical function, important [42]. Jang et al. demonstrated that thermal damage of laser etching on hip implants led to their early fracture [43]. Huber et al. [25] and Konrads et al. [26] reported a decrease in the fatigue life of Ti hip implants due to the thermal damage of electrocautery. In this context, Sonntag et al. [28] further demonstrated that this thermal damage caused a significant alteration of Ti microstructure. Ti6Al4V alloy has a bimodal microstructure consisting of a globular α-matrix phase, enveloped by the so-called Widmannstätten structure (α + β) [32,44,45,46]. Thermal energy affects the internal kinetics of theses phases and triggers a cascade of diffusion processes, as well as transforming the dimensions of the α and β phases. Alteration of those dimensions correlates with their load-to-failure and susceptibility to internal crack initiation. It could even be regarded as an internal notch [33,47,48,49].

In the light of these findings, the thermal energy applied on the tested Ti rods and the marks seen on their surface, which may be due to a corrosive attack [27] could set the stage for premature rod failure. 

### 4.2. Pathogenesis of Ti Rod Fractures at the Rod-Screw Junction

**Figure 9:**
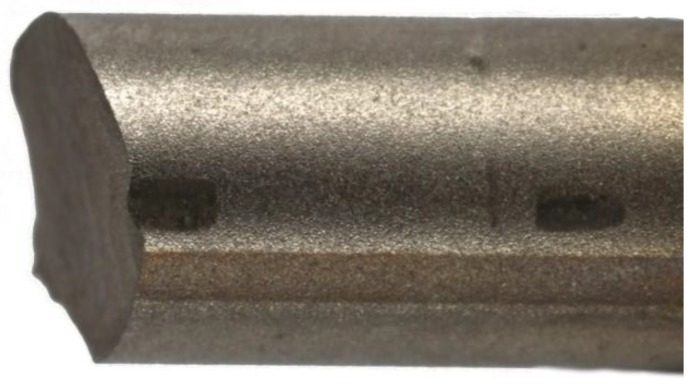
Magnified lateral view of Ti-CG post biomechanical testing: fatigue fracture of the rod at the rod-screw junction, illustrating surface marks of the tightened screws on the rod.

Among all Ti-CG and one of the Ti-EG specimens, a crack nucleation site was visually detected in the proximity of the locking screw. Tightening of the screw leaves surface marks on the rods and could account for this failure (Figure 9). The presented results echo previously published findings: Lindsey et al. and Ngyuen et al. [21,22] detected an increased trend of CoCr rod failure at the same location of our tested constructs. Yamanaka et al. [40] described the same phenomenon on Ti rods. Dick et al. [31] suggested that surgeons should avoid severe tightening of the screws as this would lead to notch formation on the surface of Ti rods. Hence, a modification of the operating manual might be indicated to avoid severe tightening of the screw. This, however, requires specific testing and is a secondary outcome of this study.

### 4.3. CoCr Versus Ti Rods

CoCr exhibited a higher fatigue life than Ti, which is in line with previous investigations [23,50,51]. However, we are not aware of previous studies addressing spinal CoCr susceptibility to thermal modification of electrocautery. Uniquely, our tested constructs were not affected by this thermal modification as the pedicle screws failed. This could be explained by their superior rigidity, corrosion, and wear resistance [52]. This biomechanical superiority is also the reason for their implementation in demanding surgical techniques such as PSO. Paradoxically, in the context of PSO, CoCr constructs were associated with higher rates of RF. This could be due to the notch effect of in situ bending inherent within the PSOs, and the unusually high loading they need to withstand [16]. However, the superior performance of CoCr comes at the expense of greater stiffness at both ends of the construct, resulting in increased incidence of adjacent segment disease [53,54,55]. This could be attributed to the fact that Ti-6Al-4V alloy has a much lower Young’s modulus (∼112 GPa) when compared to Co-Cr alloys (∼210 GPa) [56,57].

One of the strengths of our study could be its clinical implications. Patient expectations of surgical outcomes have reached unparalleled heights [58]. However, implant-related complications have significant drawbacks on post-operative Health-Related Quality of Life (HRQoL) and were associated with slower rates of improvement [1,23,59]. Moreover, the negative economic impact of instrumentation failure and post-operative complications on the health-care system in the context of spine surgery is high [60,61].

This study provides a biomechanical strongpoint by testing constructs under high sinusoidal loading levels (*n* = 8, each 10^6^ cycles) in which rod diameter was uniform, which makes the results comparable. Previous biomechanical investigations have tested constructs with different rod diameters and were tested under lower load levels [21,22]. Higher loading levels would give more confidence to the conclusion of long-term fatigue endurance. Intuitively, the longer a spinal implant persists intact in the body, the more cyclic loading it has to withstand. Testing constructs under short- and long-term mechanical loading enabled the conclusion that CoCr rods, even under long-term cyclic loading (8 × 10^6^ cycles), proved unaffected by thermal damage. Furthermore, constructs were examined according to a standard for spinal instrumentation testing [29]. The application of the ASTM F1717 standard enabled the delineation of electrocautery’s impact on the mechanical properties of materials and efficiently eliminated potential confounding factors, such as implant design or donor variability in the context of cadaver studies.

On the other hand, intrinsic to in vitro biomechanical investigations, this study was conducted within a scaffold of limitations and simplifying premises that are important to discuss. First, severity of the electrocautery attack cannot be fully controlled during manual application which could lead to heterogeneity of thermal damage on the tested rods. This could explain why the Ti-EG specimens fractured at different locations. Second, the use of only one cautery mode precludes conclusions on other electrocautery modes. Third, in vivo clinical translation of the findings is impeded by the dry conditions of these experiments which were conducted at room temperature; fatigue life could differ when implants are at body temperature and surrounded by body fluids [39]. Fourth, the application of the ASTM F1717 sinusoidal testing environment represents a “worst case scenario”; constructs alone bore the burden of the axial loading which does not fully represent the in vivo state where the load is also shared by the spine and neighboring soft tissue. However, physiological stresses on implants could be as high as 700 N which permits us to draw our conclusions, as Ti rods failed in the range of 250 N. Fifth, tested constructs underwent only axial loading, resulting in a bending moment which mimics one modality of human movement, flexion-extension, and does not take into account lateral bending and axial rotation. Finally, we did not perform a power analysis prior to conducting this study to determine the minimum sample size required for the CI of 95%. Also, the wide CI is attributed to the small sample size. However, this limitation means that the impact of electrocautery could have been underestimated in our results. Subsequently, a larger sample size would enable calculation of a statistically significant hazard ratio to quantify the negative impact of thermal damage on the fatigue life of Ti rods. Future biomechanical studies with a larger sample size are merited to analyze different settings of coagulation and cutting modes, and to assess loadings exerted by bending and axial rotation of vertebrae. Randomized controlled studies are required to determine the absolute effect of electrocautery on spinal implants. Nevertheless, our findings disentangle a potential culprit underlying post-operative Ti rod fracture.

## 5. Conclusions

This biomechanical study showed that the impact of electrocautery on Ti rods could have significant clinical and biomechanical repercussions for patient safety and satisfaction, surgical training, and implant design. Ti rods exhibited decreased fatigue life and failed at the site of electrocautery application. Spine surgeons should exercise caution in the vicinity of spinal implants, especially during revisions. Similarly, it is also reasonable for manufacturers to devise strategies against thermal damage and explore methods to increase the long-term structural integrity of Ti rods.

## Figures and Tables

**Table 1:** Specimen grouping matrix.

Group	Description	Samples
Ti-CG	Titanium rods control group (without electrocautery application)	*n* = 3
Ti-EG	Titanium electrocautery group	*n* = 3
CoCr-CG	Cobalt-Chrome control group (without electrocautery application)	*n* = 3
CoCr-EG	Cobalt-chrome electrocautery group	*n* = 3

Ti = titanium; CoCr = cobalt-chrome; CG = control group; EG = electrocautery group.

**Table 2:** Summary of the dynamic biomechanical test results.

Spinal Construct	Electrocautery Application	Completed Load Level	Min/Max Load at Failure (N)	No. Cycles to Failure	Failure Site	Fatigue Strength (N)
Ti-CG1	No	#4	30/300	**4,473,034**	Unilateral rod-screw junction	273.6
Ti-CG2	No	#4	30/300	**4,388,472**	Bilateral rod-screw junction	269.4
Ti-CG3	No	#3	25/250	**3,995,938**	Unilateral rod-screw junction	249.8
Ti-EG1	Yes	#3	25/250	**3,093,921**	Bilateral peripheral rod fractures	204.7
Ti-EG2	Yes	#3	25/250	**3,328,583**	Unilateral peripheral rod fracture *	216.4
Ti-EG3	Yes	#3	25/250	**3,770,073**	Bilateral central rod fractures	238.5
CoCr-CG1	No	#6	40/400	**6,351,621**	Bilateral pedicle screws **	367.6
CoCr-CG2	No	#7	40/400	**7,000,000**	Bilateral pedicle screws **	400
CoCr-CG3	No	#7	45/450	**7,183,433**	Bilateral pedicle screws **	409.2
CoCr-EG1	Yes	#7	45/450	**7,079,071**	Bilateral pedicle screws **	403.9
CoCr-EG2	Yes	#7	45/450	**7,118,378**	Bilateral pedicle screws **	405.9189
CoCr-EG3	Yes	#6	40/400	**6,112,167**	Bilateral pedicle screws **	355.60835

CG = Control Group; EG = Electrocautery Group; N = Newton; Ti = Titanium; CoCr = Cobalt-Chrome. * Ti-EG2 fracture site occurred at the level of the locking screws, not at the site of electrocautery application. ** all pedicle screws were made of titanium. No CoCr rods failed in this biomechanical investigation.

**Table 3:** Summary of load-to-failure and total number of cycles to failure for all tested groups.

	Load to Failure (N) *	95% CI	*p*	No. Cycles to Failure *	95% CI	*p*
**Ti-CG**	264.3 ± 12.7	[232.7–295.9]	***p*** **= 0.02**	4.3 × 10^6^ ± 25 × 10^3^	[3.6 × 10^6^–4.9 × 10^6^]	***p*** **= 0.03**
**Ti-EG**	219.8 ± 17.2	[177.2–262.5]		3.4 × 10^6^ ± 34 × 10^3^	[2.5 × 10^6^–4.2 × 10^6^]	
**CoCr-CG**	392.2 ± 21.8	[338.1–446.5]	*p* > 0.05	6.8 × 10^6^ ± 43 × 10^3^	[5.7 × 10^6^–7.9 × 10^6^]	*p* > 0.05
**CoCr-EG**	388.5 ± 28.5	[317.7–459.3]		6.8 × 10^6^ ± 57 × 10^3^	[5.3 × 10^6^–8.2 × 10^6^]	

* Mean ± standard deviation, CI = Confidence Interval, N = Newton, *p*: **Bold** denotes statistical significance, Ti = Titanium, CoCr = Cobalt-Chrome, CG = Control Group, EG = Electrocautery Group.
